# Angular limit for coronal joint deformity correction using intramedullary guidance in total knee arthroplasty. A pilot study

**DOI:** 10.1051/sicotj/2020019

**Published:** 2020-06-24

**Authors:** Chahine Assi, Jad Mansour, Camille Samaha, Pascal Kouyoumdjian, Kaissar Yammine

**Affiliations:** 1 Department of Orthopedic Surgery, Lebanese American University-Rizk Hospital, Lebanese American University School of Medicine Beirut Lebanon; 2 Department of Orthopedic Surgery, Middle East Institute of Health Bsalim Lebanon; 3 Chirurgie Orthopédique et de la Colonne Vertébrale, Centre Hospitalo-Universitaire de Nîmes Nîmes France; 4 Center for Evidence-based Anatomy, Sports & Orthopedic Research Jdeideh Lebanon

**Keywords:** Total knee arthroplasty, Coronal deformity, Knee varus, Knee valgus, Correlation

## Abstract

*Introduction*: Post-operative misalignment of the lower limb mechanical axis following total knee arthroplasty (TKA) is thought to be associated with clinical failure. In a balanced knee, a neutral global mechanical axis following the use of intra-medullary (IM) guidance does not necessarily imply a horizontal TKA joint line. Using femoral and tibial axes separately would be more accurate in evaluating TKA alignment. Thus, the aim of the study is to define a pre-operative mechanical tibial and/or femoral angle threshold value for post-operative optimal alignment correction using IM guides. *Methods*: This single-center prospective continuous pilot study included 50 patients treated with a TKA for primitive osteoarthritis. Femoral and tibial cuts were performed using intramedullary (IM) guide. Three angles were calculated and analyzed pre and post-operatively on standing antero-posterior views using long-leg radiographs: femorotibial angle (FTA), mechanical femoral angle (MFA), and mechanical tibial angle (MTA). Statistical analysis was performed for the whole sample and for the three following FTA subgroups; normo-axial, varus and valgus. *Results*: The pre-operative MTA is the only parameter for which a threshold value was observed; when pre-operative MTA exceeded the value of 94°, an optimal correction might not be obtained post-operatively. *Discussion*: Our results suggest that the bony correction obtained via IM guiding depends exclusively on the primary deformation of the tibia. In cases of a varus of more than 94°, the IM guide was found to yield sub-optimal corrections. Thus, other solutions need to be investigated.

## Introduction

One of the major long-term success elements of total knee arthroplasty (TKA) is correlated to the extent of polyethylene wear which favors loosening and consequently shortens the period for revision surgery [1, 2]. The intra-operative positioning of the prosthetic implant is considered to be by many the principal factor in predicting the extent of wear [3–5]. Many authors reported that a post-operative misalignment of the lower limb mechanical axis could lead to clinical failure with premature wear of the polyethylene [6–9]; some surgeons also use patient-specific instrumentation (PSI) to improve the accuracy of implant positioning [10].

Contrary to many earlier studies supporting TKA positioning with some degree of valgus [11, 12], a global mechanical axis within 3° has been considered till recently to be a pre-requisite to optimal TKA outcomes. It has been previously stated that residual varus of femoral origin was acceptable, but a neutral mechanical alignment of the tibial component was mandatory [13]. However, more recent reports have challenged this long-held notion of neutral mechanical alignment showing contradictory results in correlating post-operative global coronal axis to TKA optimal function and survivorship [14]. While some found no relationship between a neutral post-operative global mechanical axis and survival rate or functional outcome [15–20], others reported an increased rate of tibial components failure positioned in >3.9° of varus [9], a higher polyethylene wear in TKA aligned in >5° of varus [21], and a better survival with a valgus less than 8° [22, 23]. On the other hand, Vanlommel et al. [24] found a significantly better functional scores in knees with residual varus of 4°–7° compared to neutral and significant varus groups.

Since a global mechanical axis of 0° is not always synonymous with horizontal TKA joint line in balanced knees, the measurements of each segment, femur and tibia, separately, might be more accurate in evaluating the positioning of TKA components. Such segmental angular values might explain the discrepancy found in the literature when correlating the global mechanical axis to TKA outcomes.

This study investigated the correlation between pre- and post-operative segmental mechanical axes and would reveal different results when compared to pre- and post-operative global mechanical axes. To this, a correlation analysis was used to study the association between pre- and post-operative coronal angular values. A predictive threshold for coronal angular correction was also determined.

The aim of the study was to determine a pre-operative mechanical tibial and/or femoral angle threshold value that might hamper the post-operative correction alignment using IM guides.

## Material and methods

This is a single-center prospective pilot study conducted between March 2016 and March 2017. Ethical approval was granted from the institutional board prior to the conduct of the study. Patient consent was obtained from all included subjects. Fifty patients were included (25 males and 25 females) with a mean age of 69.1 (53–83). The population included 21 right-sided and 29 left-sided hips. Inclusion criteria were as follows: primary knee osteoarthritis, no history of previous femoral or tibial fracture, and no history of previous ligamentous injuries. Exclusion criteria were defined as revision TKA’s, ligamentous injuries, secondary diaphyseal deformation (post-traumatic sequelae, infection, mal-union), pre-operative flexion contracture, pre-operative hyperextension of more than 5°, and long-leg radiographs not appropriately taken according to the hospital standard protocol. All procedures were performed by the same senior surgeon. The tibial and femoral implant positioning was performed using only an intramedullary guide for all TKA.

### Surgical technique

In all cases, midline incision with medial parapatellar arthrotomy and lateral eversion of the patella were performed [25]. The intramedullary tibial guide used was 40 cm in length and 8 mm in width and was inserted at the midline between the tibial interconydylar eminences. The guide was shoved till the distal epiphysis of the tibia. The ancillary is set to cut perpendicular to the guide in the coronal and sagittal planes. For the femoral preparation, an intramedullary guide of 34 cm in length and 9 mm in diameter was placed 1 cm proximal to the PCL insertion and 1 cm lateral to the lateral border of the medial femoral condyle. The valgus distal cut angle was set to be the difference between the anatomical and mechanical femoral angles. Patellar resurfacing was performed in all cases. All implant components were cemented.

### Settings of radiological imaging

Pre- and postoperative long-leg radiographs were obtained using a standard protocol.

Long leg radiographs are obtained using a GE Definium 6000 Digital radiography system. The patient’s back was set toward the wall bucky, the face facing the X-ray tube, legs straight, and the feet brought together. The distance Tube-Bucky is of 180–200 cm, depending on the patient’s stature. The image acquisition procedure is automated using an Auto Image Pasting software that calculates the number of images that must be acquired to capture the entire lower limb anatomy, for a total length of 150 cm. Thus, the anterior superior iliac crest, hip joint, knee joint, tibial tubercle, and the tibio-talar (ankle) joint were included in the image.

### Definition of the knee center, axes, and mechanical angle measurements

#### Mechanical axes

The mechanical axis of the femur connects the center of the femoral head to the midline of the tangent of the distal femur.

The mechanical axis of the tibia connects the middle of the line connecting the two most proximal points of the tibial plateaus (proximal tibia) to the center of the distal tibia.

#### Mechanical angle measurements

**Figure 1:**
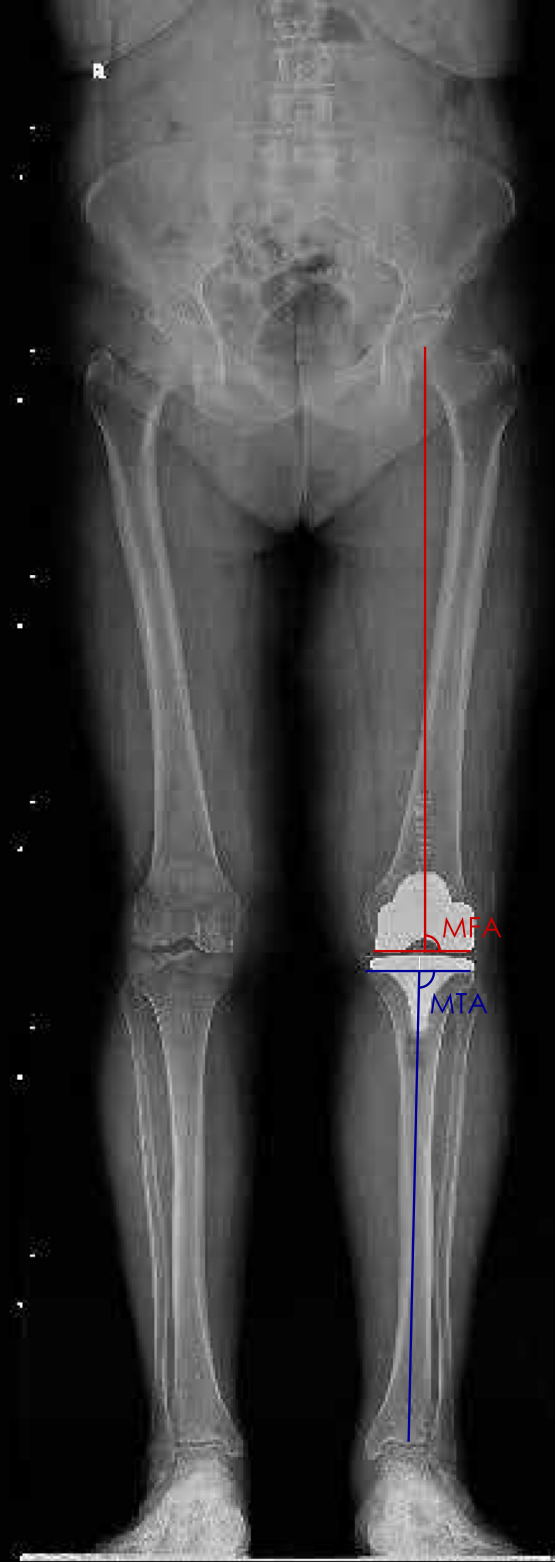
Segmental angle measurements.

The mechanical femoral angle (MFA) is defined as the lateral angle between the mechanical axis of the femur and the tangent of the distal femur (Figure 1).

The mechanical tibial angle (MTA) is defined as the lateral angle between the mechanical tibial axis and the proximal tibia (Figure 1).

The femoro-tibial angle (FTA) is defined as the angle between the femoral mechanical axis and the tibial mechanical axis. The “normal” range of FTA axis is set to be within 2° (178°–182°).

The global mechanical axis described a line drawn from the center of the femoral head to the center of the talus, usually passing through the center of the knee.

### Statistical analysis

The software StatsDirect (UK, version: 3.0.187) was used for statistical analyses.

First, inter- and intra-observer reliability analyses were conducted for each parameter using intra-class correlation coefficients (ICC) and that for each the two observers (CA and KY) and between the same observers. Excellent reliability was found as all ICC values were above 0.93.

Second, four groups were defined for correlation analysis. Group I includes all subjects, Group II includes patients with previous “normal” angles (178° < FTA < 182°), Group III includes subjects with pre-operative global axis in varus (FTA > 182°), and Group IV includes those with a pre-operative global axis in valgus (FTA < 178°). A spearman test was used to look for potential correlations between pre- and post-operative mechanical angular values. The receiver operating characteristic (ROC) curve was used to determine a threshold for correction.

## Results

Nine patients with an average age of 68 years presented with pre-operative valgus with an average angle of 7.25°. Thirty-five patients with an average age of 70 year (53–83) presented with pre-operative varus with an average angle of 8°. Six patients with an average age of 69 years had normo-axial knees.

Table 1 summarizes the pre and post-operative angle values. Table 2 shows the correlation analysis results and the ROC values.

**Table 1:** Patients’ demographics and pre-and post-operative angular values.

Sex	Age op.	Side	MFA pre	MFA post	MTA pre	MTA post	FTA pre	FTA post	FTA theor.
Normo-axis (6 patients)									
F	77	L	83	91	93	90	179	181	181
M	63	R	88	90	86	92	179	183	182
M	82	R	88	90	94	94	179	183	184
F	74	R	88	90	93	91	180	181	181
F	78	R	89	90	95	90	181	182	180
M	58	R	85	88	95	90	181	177	178
Valgus (9 patients)									
F	69	L	87	88	88	90	168	177	178
F	65	L	85	89	85	89	169	177	178
F	70	L	82	91	93	91	172	183	182
F	62	R	83	90	93	90	173	178	180
F	65	R	85	90	92	93	173	183	183
M	54	L	88	90	95	89	174	179	179
F	66	L	85	84	91	90	175	173	174
F	72	R	88	89	88	90	178	179	179
M	75	L	89	91	93	91	178	181	182
Varus (35 patients)									
F	72	L	89	90	90	89	182	178	179
M	71	R	90	90	94	94	182	183	184
F	66	R	85	90	92	90	183	179	180
F	75	L	88	86	92	90	184	186	176
M	63	L	90	90	95	90	184	181	180
M	56	R	87	93	97	93	184	186	184
F	72	L	86	88	104	93	184	181	181
M	69	L	89	88	95	94	185	182	182
M	80	R	89	90	92	86	185	176	176
M	75	L	88	92	95	92	185	184	184
M	65	L	89	86	95	92	185	178	178
F	68	L	87	88	94	93	185	181	181
M	67	R	88	88	97	92	186	180	180
M	69	L	88	89	98	91	186	180	180
M	69	L	88	87	94	92	186	178	179
F	64	L	86	88	96	92	186	178	180
M	75	R	88	87	95	93	187	180	180
M	81	R	88	90	95	91	187	181	181
M	53	R	96	92	98	93	187	185	185
M	74	L	90	90	94	91	187	181	181
F	66	L	89	88	98	93	188	182	181
F	83	L	94	91	93	90	189	181	181
F	69	L	93	91	92	92	189	184	183
M	64	L	90	89	92	90	189	177	179
F	68	R	92	92	96	92	189	184	184
F	75	R	88	88	96	95	189	183	183
F	69	R	90	88	93	93	190	179	181
M	66	L	90	94	98	92	191	185	186
M	74	L	88	87	98	92	191	178	179
F	73	L	90	93	94	93	192	195	186
M	68	R	94	92	94	90	192	181	182
F	59	L	94	88	94	90	195	176	178
M	71	L	95	93	98	90	196	186	183
M	57	R	90	90	100	90	200	181	180
F	78	L	95	90	102	92	201	181	181

**Table 2:** Correlation results and ROC values.

Deformity	Angle of study	Correlation *R*	CI	DF	Double-sided *P*	ROC value
Varus subgroup	MFA	0.44	0.123 to 0.673	33	0.008*	–
	MTA	0.35	0.011 to 0.400	33	0.038*	94°
	FTA	0.11	−0.190 to 0.372	33	0.51	–
Valgus subgroup	MFA	0.005	−0.661 to 0.667	7	0.99	–
	MTA	0.29	−0.458 to 0.802	7	0.43	92°
	FTA	0.14	−0.577 to 0.735	7	0.72	–
Normal subgroup	MFA	−0.01	−0.816 to 0.806	4	0.98	–
	MTA	−0.28	−0.890 to 0.686	4	0.58	93°
	FTA	−0.62	−0.952 to 0.380	4	0.18	–
Total sample	MFA	0.32	0.047 to 0.550	48	0.02*	–
	MTA	0.36	0.093 to 0.581	48	0.009*	94°
	FTA	0.27	−0.008 to 0.510	48	0.057	–

The statistical analysis revealed a significant correlation between MFA and MTA angular values pre and post-operatively only for the varus group (*r* = 0.008 and 0.038, respectively).

**Figure 2:**
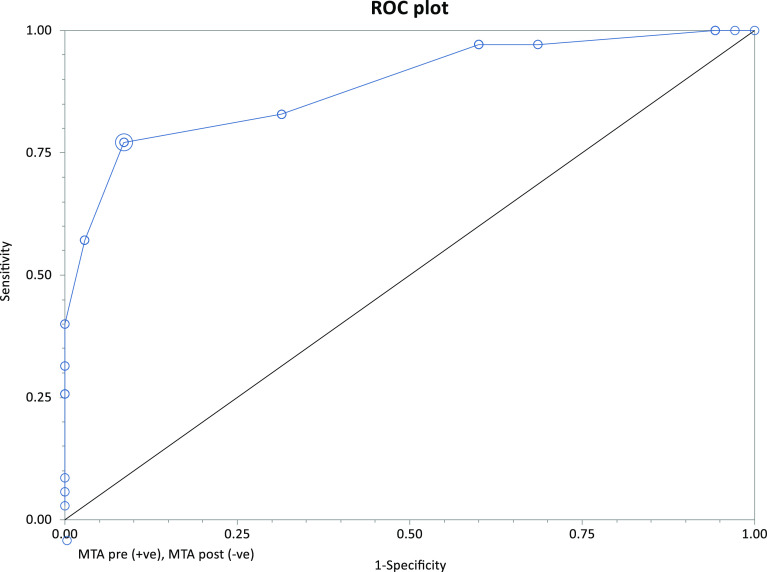
ROC curve analysis.

The search for a threshold level for correction was found for the MTA only (Area under ROC = 0.886, 95% CI = 0.809–0.962). The optimum cut-off point selected was 94°; this value expressed the maximal limit for a surgical correction (MTA < 92°) of the tibial component in the coronal plane (Figure 2).

## Discussion

The findings of this study indicate that the tibial component positioning is difficult to achieve using IM guiding when pre-operative epiphyseal tibial varus exceeded 4°.

The literature associated to the impact of coronal alignment on TKA function and survival was found to be inconclusive. Many earlier studies [12, 26–28] presented better clinical results associated with geometric TKA anatomically positioned with some degree of valgus. While Townley [29] claimed that the global mechanical axis should lie medial to the center of the knee, in varus-aligned knees, Insall et al. [11] believed that the axis must produce a valgus alignment. Most of these earlier studies used short-leg radiographs and first generations component designs.

Subsequent studies using long-leg radiographs, supported the restoration of the mechanical axis to 0° (SD ± 3°) [30–33], a concept popularized by Werner et al. (1991) [33]. However, more recent studies showed contradictory conclusions over the relationship between global TKA alignment and survivorship. Parratte et al. [17] and Bonner et al. [18] found no relationship between a post-operative mechanical axis of 0° (SD ± 3°) and a 15-year survival rate. Matziolis et al. [15] found no difference in durability or outcome between aligned TKAs and a subset of varus groups. Similarly, Morgan et al. [16] reported no statistical significance between TKA survivorship and the anatomical femoral-tibial angle. However, in a review performed by Abdel et al., they found that a neutral mechanical axis remains the optimal guide of alignment [34].

On the other hand, Berend et al. [9] reported a statistically increased rate of tibial components failure when positioned in >3.9° of varus. Fang and Ritter [22] found a better survival with an overall anatomical alignment of between 2.4° and 7.2° of valgus. Ritter et al. [23] found that an increased rate of failure was associated with (a) a femoral component in >8° of anatomical valgus, (b) a varus tibial component relative to the tibial axis and (c) when one component was positioned to compensate for the other component malalignment. Furthermore, a significantly greater loss of polyethylene thickness in the medial compartment was found in TKA aligned in >5° of varus [21].

Additionally, the functional outcomes of kinematically aligned TKAs are still unclear. Howell et al. [35] showed no improvement in the functional score in relation to lower limb alignment while Dossett et al. [36] found a significant difference in favor of kinematically aligned TKAs. In a study performed by Lording et al. (2016), the authors found that a mild global varus deformity my not affect global outcomes but survivorship of the implant may have a negative impact if the tibial components is positioned in varus [37].

This study could have the potential to explain such contradictory results. We believe that segmental mechanical angular values would be better indicators for the assessment of the femoral and tibial component positioning than the FTA. To this and instead of taking the global mechanical axis as the reference for evaluating the post-operative TKA coronal positioning, we calculated the pre- and post-operative mechanical tibial and mechanical femoral axis separately in order to compute the correlation before and after the surgery. In fact, though a positioning of each implant at an angle of 90° would avoid later complications in a balanced knee, a global mechanical axis of 180° does not necessarily mean an optimal horizontal TKA line. An oblique TKA joint line in a balanced knee, whether in varus or valgus, might be present with a global mechanical axis of 180°. Calculating separately the mechanical axis of each segment, femoral and tibial, would be more accurate in evaluating the positioning of each implant, provided that the knee is balanced. Our results demonstrated that the femoral positioning is typically correctly achieved (≤2°) using an intramedullary guide. On the other hand, the tibial component positioning was found to be seldom optimal when the initial varus deformity was higher than 4°. In such cases, an optimal positioning of the tibial implant cannot be achieved using a 90° intramedullary guided cut. This could explain the reason behind the low functional scores and survivorship reported in TKAs with residual postoperative varus deformity caused by tibial component placed in varus [19]. Moreover, among patients with at least 10° of preoperative varus, these same authors found better functional scores in patients with residual varus compared to those with neutral alignment. Our study would indicate that such confusing results are due the reference axis of study. In fact, all reported literature in relation to TKA results is based on the global mechanical axis. We believe that the global mechanical axis is not the appropriate axis to be used in evaluating TKA component positioning and functional survival outcomes. Our study indicates that segmental axes, mainly the tibial mechanical axis, provide better references for assessment of TKA results. For instance, a recent review concluded that residual varus alignment might not adversely impact survivorship provided that the tibial component is positioned in neutral alignment [37]. In this study, if the mechanical tibial axis was used as the reference rather than the global one, functional and survivorship outcomes would have been more explicit for interpretation.

The superiority of one guided cut method over the other is still not demonstrated. Lotke and Ecker proved an advantage in using intramedullary guides in comparison to extra medullary guides (88% of orthogonal cuts against 70%) [26], confirming the results obtained by Engh and Petersen (94% against 85%) [38] In contrast, Baldini et al. [39] revealed opposite results, giving the superiority to the use of an extra-medullary guide ancillary. Hungerford and Krackow reported that the femoral component was more likely to be placed in flexion position using IM system, while both extra and intramedullary guidance were found to similarly improve accuracy of tibial component positioning [40]. Again all those studies used the global axis as the reference axis and none of these investigated a threshold value of correction. Computer assisted guidance for implant positioning has been presented as an advanced solution [41]; a recent meta-analysis displayed better short term functional outcomes when using imageless computer navigation [42–44], but without being necessarily correlated with an optimal global alignment. In addition, patient-specific instrumentation (PSI) has been presented to improve accuracy in TKA but there utility is still controversial [45]. In a study performed by Jenny et al. found that in a randomly selected population of 100 subjects with no known knee abnormalities, they were found to have a wide variation in lower limb axes, thus concluding that the use of individual personalized instruments and implants might provide a possible alternative to standard TKA’s [46].

The limitations of this study are mainly correlated to its small sample size. However, we have acknowledged this study as a pilot study and a prospective protocol has been prepared to confirm the statements using a much larger sample. Future studies are needed to investigate the correlation between segmental axis values and TKA survivorship and functional outcomes. Additionally, the number of patients in the valgus group is considered as insufficient to identify any difference. This latter limitation reflects the low prevalence of genu valgum knees detected in the general population.

## Conclusion

In this study, we quantified a threshold value of 94° for the pre-operative tibial varus deformity using intramedullary guide above which optimal TKA mechanical alignment is unlikely to be obtained. When assessing TKA positioning, we believe that the use of segmental mechanical angles could be more accurate than the global mechanical angle. That could explain the inconsistency reported in many studies when analyzing the association between the global mechanical axis and TKA clinical outcomes.

## Conflict of interest

The authors declare that they have no conflict of interest.
